# Detection and Genome Sequencing of SARS-CoV-2 Variants Belonging to the B.1.1.7 Lineage in the Philippines

**DOI:** 10.1128/MRA.00219-21

**Published:** 2021-05-06

**Authors:** Francis A. Tablizo, Cynthia P. Saloma, Marc Jerrone R. Castro, Kenneth M. Kim, Maria Sofia L. Yangzon, Carlo M. Lapid, Benedict A. Maralit, Marc Edsel C. Ayes, Jan Michael C. Yap, Jo-Hannah S. Llames, Shiela Mae M. Araiza, Kris P. Punayan, Irish Coleen A. Asin, Candice Francheska B. Tambaoan, Asia Louisa U. Chong, Karol Sophia Agape R. Padilla, Rianna Patricia S. Cruz, El King D. Morado, Joshua Gregor A. Dizon, Eva Maria Cutiongco-de la Paz, Alethea R. de Guzman, Razel Nikka M. Hao, Arianne A. Zamora, Devon Ray Pacial, Juan Antonio R. Magalang, Marissa Alejandria, Celia Carlos, Anna Ong-Lim, Edsel Maurice Salvaña, John Q. Wong, Jaime C. Montoya, Maria Rosario Singh-Vergeire

**Affiliations:** a Core Facility for Bioinformatics, Philippine Genome Center, University of the Philippines System, Quezon City, Philippines; b Philippine Genome Center, University of the Philippines System, Quezon City, Philippines; c DNA Sequencing Core Facility, Philippine Genome Center, University of the Philippines System, Quezon City, Philippines; d Clinical Genomics Laboratory, Philippine Genome Center, University of the Philippines System, Quezon City, Philippines; e Epidemiology Bureau, Department of Health, Manila, Philippines; f Disease Prevention and Control Bureau, Department of Health, Manila, Philippines; g Inter-Agency Task Force on Emerging Infectious Diseases (IATF) Technical Working Group on COVID-19 Variants, Department of Health, Manila, Philippines; DOE Joint Genome Institute

## Abstract

We report the sequencing and detection of 36 severe acute respiratory syndrome coronavirus 2 (SARS-CoV-2) samples containing lineage-defining mutations specific to viruses belonging to the B.1.1.7 lineage in the Philippines.

## ANNOUNCEMENT

Coronavirus disease 2019 (COVID-19) is an infectious disease that has gained pandemic status from the World Health Organization, with millions of cases and deaths recorded worldwide. This global health crisis is caused by the virus referred to as severe acute respiratory syndrome coronavirus 2 (SARS-CoV-2), a member of the genus *Betacoronavirus* (*Coronaviridae*), together with the causative agents of the first SARS outbreak in 2003 and the Middle East respiratory syndrome (MERS) in 2012.

In this study, we present the genome sequences of 36 cases of COVID-19 in the Philippines caused by viruses belonging to SARS-CoV-2 lineage B.1.1.7, also referred to as 20I/501Y.V1 or the variant of concern (VOC) 202012/01. This particular SARS-CoV-2 variant was initially identified in the United Kingdom and has been reported to cause a surge of COVID-19 infections in that country (1). Initial studies also suggest that the B.1.1.7 viruses appear to have a replicative advantage (2) and are more transmissible (3). The protocols used in this study were reviewed and approved by the Single Joint Research Ethics Board of the Department of Health, with approval code SJREB-2021-11, as part of a larger research program entitled “A retrospective study on the national genomic surveillance of COVID-19 transmission in the Philippines by SARS-CoV-2 genome sequencing and bioinformatics analysis.”

In order to detect the entry of B.1.1.7 infection into the Philippines, nasopharyngeal swabs were collected between 10 December 2020 and 31 January 2021 from COVID-19 cases detected in returning overseas Filipinos, as well as from local case clusters, mainly from the Cordillera Administrative Region of the country, among others. Only reverse transcriptase PCR (RT-PCR)-positive cases with a cycle threshold (C_*T*_) value below 30 in any gene target were considered for the subsequent sequence analysis. The collected swab samples were then subjected to RNA extraction using the QIAamp viral RNA minikit, the product of which was used as the template for the amplicon-based Illumina COVIDSeq test sequencing workflow.

The resulting sequence reads were mapped to the reference SARS-CoV-2 genome (NCBI accession number NC_045512.2) using minimap2 version 2.17-r941 (4), with the “–x sr” parameter for accurate genomic short-read alignment. Primer clipping and quality trimming, intrahost variant calling, removal of reads associated with mismatched primer indices, and consensus sequence assembly were then performed following the suggested workflow of iVar version 1.2.2 (5), using default parameters. The consensus variants were identified by comparing the resulting assemblies with the reference sequence using MUMmer (6), as implemented in RATT software (7). Lastly, SARS-CoV-2 lineage classifications (8) were assigned using PANGOLIN version 2.3.2 (https://github.com/cov-lineages/pangolin).

A total of 36 Philippine SARS-CoV-2 samples were classified under the B.1.1.7 lineage. Table 1 shows the primary consensus assembly metrics for these samples. The average depth of coverage across all the sequences is 1,183×, with 26 of the samples carrying all 17 hallmark mutations of the B.1.1.7 lineage as listed in the PANGO lineages report for the B.1.1.7 variant of concern (https://cov-lineages.org/global_report_B.1.1.7.html).

**TABLE 1:** Primary consensus sequence assembly metrics

Sample code	NCBI accession no. for:		Collection date (day mo yr)	% GC content	Sample source^*a*^	Mean coverage depth (×)	No. of consensus SNPs^*b*^	No. of B.1.1.7 SNPs	% N^*c*^	Length (bp)
	GenBank	SRA								
PH-PGC-00315	MW735407	SRR13907363	29 Dec 20	37.32	ROF	1,201.27	52	17	1.76	29,884
PH-PGC-00317	MW735408	SRR13907362	29 Dec 20	37.48	ROF	1,194.12	50	17	1.30	29,884
PH-PGC-00401	MW735409	SRR13907351	10 Dec 20	37.22	ROF	996.07	51	17	2.12	29,884
PH-PGC-00986	MW735410	SRR13907340	7 Jan 21	37.14	ROF	1,164.12	50	17	2.26	29,884
PH-PGC-02005	MW735411	SRR13907330	4 Jan 21	31.86	CAR	508.19	44	14	16.82	29,884
PH-PGC-02008	MW735412	SRR13907329	3 Jan 21	35.45	CAR	740.89	48	17	6.87	29,884
PH-PGC-02009	MW735413	SRR13907328	3 Jan 21	34.31	CAR	689.44	45	14	10.19	29,884
PH-PGC-02033	MW735414	SRR13907327	5 Jan 21	37.38	CAR	1,362.04	49	17	1.52	29,884
PH-PGC-02127	MW735415	SRR13907326	7 Jan 21	33.83	CAR	973.77	45	14	11.36	29,884
PH-PGC-02131	MW735416	SRR13907325	7 Jan 21	36.53	CAR	1,326.01	49	17	3.77	29,885
PH-PGC-02133	MW735417	SRR13907361	7 Jan 21	34.19	CAR	892.30	49	16	10.20	29,884
PH-PGC-02152	MW735418	SRR13907360	9 Jan 21	36.16	CAR	1,086.44	38	16	4.80	29,884
PH-PGC-02181	MW735419	SRR13907359	8 Jan 21	36.40	CAR	882.19	50	17	4.18	29,884
PH-PGC-02183	MW735420	SRR13907358	8 Jan 21	37.06	CAR	1,210.23	51	17	2.42	29,884
PH-PGC-02184	MW735421	SRR13907357	8 Jan 21	35.20	CAR	891.29	48	16	7.54	29,884
PH-PGC-02185	MW735422	SRR13907356	8 Jan 21	37.33	CAR	1,131.33	49	17	1.69	29,884
PH-PGC-02225	MW735423	SRR13907355	8 Jan 21	37.13	CAR	1,303.29	51	17	2.14	29,884
PH-PGC-02408	MW735424	SRR13907354	7 Jan 21	37.92	ROF	1,652.89	51	17	0.10	29,884
PH-PGC-02434	MW735425	SRR13907353	12 Jan 21	37.70	ROF	1,168.26	53	17	0.78	29,884
PH-PGC-02630	MW735426	SRR13907352	16 Jan 21	37.83	ROF	1,307.23	48	17	0.41	29,884
PH-PGC-02725	MW735427	SRR13907350	14 Jan 21	37.16	ROF	1,140.03	52	17	2.04	29,884
PH-PGC-02730	MW735428	SRR13907349	16 Jan 21	37.87	ROF	1,620.34	52	17	0.13	29,884
PH-PGC-02732	MW735429	SRR13907348	17 Jan 21	37.89	ROF	724.58	52	17	0.25	29,884
PH-PGC-02733	MW735430	SRR13907347	17 Jan 21	37.88	ROF	1,499.29	51	17	0.15	29,884
PH-PGC-02745	MW735431	SRR13907345	19 Jan 21	30.64	ROF	679.99	32	12	19.71	29,894
PH-PGC-02756	MW735432	SRR13907344	15 Jan 21	37.01	CAR	1,037.94	50	17	2.23	29,884
PH-PGC-02770	MW735433	SRR13907343	15 Jan 21	35.34	ROF	971.34	44	14	6.34	29,885
PH-PGC-02793	MW735434	SRR13907342	19 Jan 21	37.65	ROF	1,290.90	50	17	0.90	29,884
PH-PGC-02812	MW735435	SRR13907341	24 Jan 21	37.91	CAR	1,654.09	50	17	0.18	29,884
PH-PGC-02826	MW735436	SRR13907339	21 Jan 21	37.85	CAR	1,346.54	52	17	0.31	29,884
PH-PGC-02845	MW735437	SRR13907338	13 Jan 21	37.74	CAR	1,454.34	51	17	0.58	29,884
PH-PGC-02851	MW735438	SRR13907337	11 Jan 21	37.87	CAR	1,563.14	49	17	0.28	29,884
PH-PGC-02886	MW735439	SRR13907336	16 Jan 21	37.67	CAR	1,411.43	51	17	0.86	29,884
PH-PGC-03846	MW735440	SRR13907334	24 Jan 21	37.87	ROF	1,458.88	49	17	0.16	29,884
PH-PGC-03939	MW735441	SRR13907333	31 Jan 21	37.89	ROF	2,082.45	50	16	0.27	29,885
PH-PGC-03978	MW735442	SRR13907332	25 Jan 21	37.25	NCR	990.68	53	16	2.01	29,884

a ROF, returning overseas Filipino; CAR, Cordillera Administrative Region; NCR, National Capital Region.

b SNPs, single nucleotide polymorphisms.

c % N, percentage of ambiguous base calls (“N” content) in the consensus sequence assembly. High percent N values generally result in lower percent GC content.

The detection of B.1.1.7 from returning overseas Filipino workers and in the community highlights the need for genomic surveillance at the country’s ports of entry and in the general population to monitor the importation and local transmission of emerging variants of concern that may impact the public health response to the SARS-CoV-2 pandemic in the Philippines.

### Data availability.

The consensus sequence assemblies reported in this study have been deposited in the NCBI GenBank database, and their corresponding read alignments (BAM format) are in the NCBI Sequence Read Archive (SRA) database under BioProject accession number PRJNA708134. The accession numbers for the GenBank and SRA submissions are provided in Table 1.
